# Activation and Evasion of RLR Signaling by DNA Virus Infection

**DOI:** 10.3389/fmicb.2021.804511

**Published:** 2021-12-20

**Authors:** Junli Jia, Jiangan Fu, Huamin Tang

**Affiliations:** ^1^Department of Immunology, Nanjing Medical University, Nanjing, China; ^2^Genor Biopharma Co., Ltd., Shanghai, China; ^3^Laboratory Center for Basic Medical Sciences, Nanjing Medical University, Nanjing, China; ^4^Key Laboratory of Antibody Technique of Ministry of Health, Nanjing Medical University, Nanjing, China

**Keywords:** RIG-I-like receptor, RNAPIII, MAVS, MDA5, LPG2

## Abstract

Antiviral innate immune response triggered by nucleic acid recognition plays an extremely important role in controlling viral infections. The initiation of antiviral immune response against RNA viruses through ligand recognition of retinoic acid-inducible gene I (RIG-I)-like receptors (RLRs) was extensively studied. RLR’s role in DNA virus infection, which is less known, is increasing attention. Here, we review the research progress of the ligand recognition of RLRs during the DNA virus infection process and the viral evasion mechanism from host immune responses.

## Introduction

The innate immune response is the first line of host defense against microbial infection (Zhao and Karijolich, 2019). Pattern-recognition receptors (PRRs) detect pathogen-associated molecular patterns (PAMPs) structures, most of which, if not all, are crucial for their life cycle. Pathogen-specific sequences of DNA and RNA present in viral, bacterial, and eukaryotic genomes are one of the important categories of PAMPs detected by host PRRs (Goubau et al., 2013; Liu and Gack, 2020). A set of nucleic-acid-detecting PRRs have been identified, including Toll-like receptors (TLRs), RIG-I-like receptors, NOD-like receptors (NLRs), and other cytosolic and nucleic sensors. Some are located in intracellular compartments, and others are in the cytoplasm, nucleus, or cellular membrane (Ablasser and Sun, 2019). RLRs recognize RNA virus genomes and/or their products, which plays an important role in initiating the antiviral innate immune response against various RNA viruses (Yoneyama et al., 2004; Rehwinkel and Gack, 2020). Interestingly, activation and downstream signal transduction of RLRs have also been recently observed during several DNA viruses’ infections (Andrejeva et al., 2004; Gack et al., 2009; Ban et al., 2018; Koliopoulos et al., 2018; Zhang et al., 2020; Jiang et al., 2021).

## RLR Discovery and Their Structure

Three members of the RLR family, RIG-I (also named as DDX58), MDA5 (melanoma-differentiation-associated gene 5), and LGP2 (laboratory of genetics and physiology 2), have been identified to be expressed in most types of cells and tissues (Yoneyama et al., 2005).

RIG-I was originally identified as a retinoic acid-treated cell induction gene from acute promyelocytic leukemia (Liu et al., 2000). Subsequently, porcine RIG-I homologs were identified as porcine reproductive and respiratory syndrome virus-induced genes, suggesting a possible association between RIG-I and viral infection (Zhang et al., 2000). Later studies have shown that RIG-I interacts with intermediate proteins called MAVS (mitochondrial antiviral signaling; IPS-1/VISA/Cardif) during downstream signal transduction, ultimately leading to activation IRF3/7 (interferon regulatory factors 3 and 7) and/or NF-κB transcription factors (Kawai et al., 2005; Meylan et al., 2005; Seth et al., 2005; Xu et al., 2005), which results in the production of type I interferons (IFNs) and other proinflammatory cytokines (Wu et al., 2013; Peisley et al., 2014; Onomoto et al., 2021).

MDA5 is the closest relative of RIG-I, exhibiting 23 and 35% sequence similarities in the N-terminal and C-terminal domains, respectively (Kang et al., 2002). MDA5 was identified as an upregulated gene in a human melanoma cell line, HO-1 cells, after combined treatment with IFN-β and MEZ (an anti-leukemic PKC activating compound that induces terminal differentiation). Thus, initially, it was thought to be a type I interferon-responsive apoptosis-inducing gene (Kang et al., 2004). Later, MDA5 was found as a binding target for V proteins of paramyxoviruses (simian virus 5, human parainfluenza virus 2, mumps virus, Sendai virus, and hendra virus). Since V proteins inhibit the dsRNA-induced activation of the IFN-induced genes, this indicates MDA5 has a role in antiviral immune responses (Andrejeva et al., 2004; Rodriguez and Horvath, 2014).

LGP2 has 31 and 41% sequence similarities with the helicase domains of RIG-I and MDA5, respectively (Rothenfusser et al., 2005; Yoneyama et al., 2005; Komuro and Horvath, 2006). Expression of LGP2 inhibits the signaling to IFN-stimulated regulatory element (ISRE)- and NF-κB-dependent pathways induced by infection with Sendai virus and Newcastle disease virus (Rothenfusser et al., 2005). LGP2 can compete with the kinase IKK-*i* for a common interaction site on MAVS, inhibiting NF-κB-dependent signaling pathway activation. Thus, LGP2 is proposed as a regulatory element of intracellular antiviral signaling (Komuro and Horvath, 2006).

All three RLRs are members of the nucleic acid-dependent NTPases superfamily 2 (SF2) and share two structure similarities, a central helicase domain (responsible for intrinsic dsRNA binding and ATP hydrolysis functions) and a carboxy-terminal domain (CTD; responsible for binding to RNA termini) which are involved in the detection of immunostimulatory RNAs (Luo et al., 2011, 2013). Only RIG-I and MDA5 have two caspase activation and recruitment domains (CARDs) in their N-terminal region, which are responsible for downstream signal transduction. In uninfected cells, RIG-I CARDs are found in a resting state by folding with the helical insertion domain in its helicase domains (Kowalinski et al., 2011), whereas MDA5 activity is repressed by phosphorylation (Wies et al., 2013). After recognition and binding to immunostimulatory RNA, RLRs hydrolyze ATP and change their conformation, resulting in the release of CARDs for MAVS interactions and signal transduction (Myong et al., 2009; Peisley et al., 2013; Devarkar et al., 2018). LGP2 lacks the CARDs and is commonly known to regulate RIG- I and MDA5 (Kato et al., 2008; Schlee et al., 2009; Schmidt et al., 2009; Duic et al., 2020).

## Ligand Recognition by RLRs

Since host RNAs are present together with pathogen RNAs in the cytosol of infected cells, RLRs need to discriminate immunostimulatory pathogen RNAs from physiologically normal RNAs. Cellular mRNAs, often single-stranded and 5′-triphosphorylated, acquire a cap structure involving 2′-O- methylation (cap 1 or cap 2) in the cell nucleus following transcription (Zuest et al., 2011; Schuberth-Wagner et al., 2015). Typical 28S and 18S ribosomal RNAs (rRNAs) and tRNAs have only a single phosphate at the 5′ end. Although 5S rRNA contains a 5′-PPP moiety, it is methylated and complexed with proteins (Chiang J.J. et al., 2018). These host RNAs normally do not have features required for RLR recognition. RNA structures recognized by RIG-I are relatively well elucidated. These RNAs are double-stranded, with 5′−triphosphate or diphosphate, and either have an incompletely methylated cap structure (cap 0) (Decroly et al., 2012) or are bearing a protein covalently attached to the 5′ end (such as VPg) (Cardenas et al., 2006). It has also been reported that single-strand viral genomes possessing 5′-phosphorylated feature (without cap structure) can trigger RIG-I/MAVS signaling (Hornung et al., 2006; Pichlmair et al., 2006). Ren et al. (2019) recently reported that 5′-monophosphate RNAs block RIG-I upon binding to the RIG-I CTD domain.

The sensing and activation of RIG-I like receptors are not well understood, although much work has been done to elucidate RNA-triggered MDA5 activation. MDA5 forms filaments with a ring-like conformation around dsRNA in a sequence-non-specific manner (Berke and Modis, 2012; Peisley et al., 2012). In addition, long poly I: C molecules preferentially trigger MDA5, whereas short poly I: C ones activate RIG (Kato et al., 2008; Berke and Modis, 2012; Wu et al., 2013). Therefore, now it is commonly accepted that MDA5 detects long dsRNA and their secondary structure.

The mechanisms by which LGP2 regulates RIG-I/MDA5-mediated signaling remain to be further investigated (Li et al., 2009; Uchikawa et al., 2016). Both positive and negative regulatory function of LGP2 was reported (Uchikawa et al., 2016). *In vitro* studies showed that LGP2 could affect dsRNA recognition and MAVS interaction of RIG-I and MDA5 and compete with IKK-*i* for recruitment to MAVS (Komuro and Horvath, 2006; Saito et al., 2007). Lgp2^–/–^ mice and mice harboring a point mutation in the LGP2 helicase domain (K30A) showed that LGP2 functions as a positive regulatory factor and enhances RIG-I- and MDA5-mediated antiviral activity (Satoh et al., 2010). Furthermore, by using biochemical and biophysical approaches, LGP2 was found to be incorporated into the fibers of MDA5, promoting the exposure of its CARDs, resulting in a change to active conformation and signaling activity (Kato et al., 2006; Bruns et al., 2014; Duic et al., 2020).

## Modulation of RLRs Activation

Innate immune signaling is tightly controlled to prevent excessive inflammatory responses. Post-translational modifications (PTMs) play an important role during this process, including phosphorylation, ubiquitination, ISGylation, acetylation, and other less well-studied modifications (Du et al., 2018). Among them, ubiquitination modification is extensively investigated.

TRIM25 is the first E3 ligase identified for CARD-mediated signaling activation of RLG-I. Subsequent studies also showed that modification of RIG-I by TRIM25 is also influenced by several other host factors (Gack et al., 2007). The mitochondrial-targeting chaperone 14-3-3ε forms a complex with RIG-I and TRIM25 and regulates their transition from the cytoplasm to the mitochondrial (Liu et al., 2012). Cyclophilin A enhances the interaction between RIG-I and TRIM25 during the infection of some viruses, such as Sendai virus. NDR2 directly interacts with RIG-I and TRIM25 and promotes the K63-linked polyubiquitination of RIG-I mediated by TRIM25 (Liu et al., 2019). TRIM25 also interacts with NLRP12 resulting in disrupting Lys63 ubiquitination and activation of RIG-I (Chen et al., 2019). Other E3 ligases like TRIM4 and MEX3C (Mex-3 RNA binding family member C) have also been shown to conjugate covalent K63-linked polyubiquitin chains to RIG-I and mediate its activation (Davis and Gack, 2015). Alternatively, RIG-I also undergoes K48-linked ubiquitination by RNF125, RNF122 (ring finger protein 125) (Jia et al., 2017) and c-Cbl (Casitas B-lineage lymphoma proto-oncogene) (Chen et al., 2013) for proteasomal degradation to form a negative feedback loop of RIG-I signaling. Ubiquitination-dependent signaling was also reported in MDA5 activation. TRIM65 is responsible for K63-linked ubiquitination at Lys743 of MDA5 and arrestin domain containing 4 (ARRDC4) interacts with MDA5 to recruit the E3 ligase (Lang et al., 2017).

Several deubiquitinating enzymes have been demonstrated to function in the RLRs activation process. If the K48-linked ubiquitin chains are eliminated, it will promote signaling activation, such as the function of ubiquitin specific peptidase 4 (USP4) and ovarian tumor domain-containing ubiquitin aldehyde binding protein 1 (OTUB1) (Wang et al., 2013a; Jahan et al., 2020). Conversely, multiple proteins have been identified to deubiquitinate K63-linked ubiquitin from RLRs, which results in signaling inhibition (Friedman et al., 2008; Cui et al., 2014; Fan et al., 2014; Lin et al., 2016; Tao et al., 2020).

Phosphorylation of RLRs (extensively investigated in RIG-I and MDA5) mainly shows inhibitory effects on their activation. Phosphorylation on CARDs (at S8 and T170) and the CTD (at T770 and S854/855) of RIG-I and on MAD5 (at S88 and S828) suppresses the activation of these sensors, if not all, most of which are removed during their activation (Gack et al., 2010; Nistal-Villan et al., 2010; Sun et al., 2011). Dynamic sumoylation and desumoylation of MDA5 and RIG-I have also been reported. In uninfected cells or at the early stage of infection (Sendai virus infection), the sumoylation of MDA5 and RIG-I suppresses their K48-linked polyubiquitination and degradation. At the late phase of the viral infection, the modification is removed by a host factor, SENP2 (sentrin/sumo-specific protease 2), which results in their K48-linked polyubiquitination and degradation of these RLRs (Hu et al., 2017). ISG15 (IFN-stimulated gene 15) is a small ubiquitin-like protein that could be covalently conjugated onto RLRs, and this modification (ISGylation) also contributes to activation of the sensors (Ketscher et al., 2015; Villarroya-Beltri et al., 2017; Zhang et al., 2017; Perng and Lenschow, 2018). Just like ubiquitination, ISGylation is also a reversible process that could be removed by ubiquitin-specific peptidase 18 (USP18) protein (Malakhov et al., 2002; Ritchie et al., 2017).

## DNA Virus Detection by RLRs

Functions of the cytoplasmic DNA sensor, cyclic GMP-AMP synthase (cGAS) during the infection of several DNA viruses have been exclusively investigated (Hopfner and Hornung, 2020). Interestingly, in some cases of DNA virus infection, stimulation of innate immune reaction also depends on RLR/MAVS signaling activation (Chan and Gack, 2016). The most intriguing question about this phenomenon was how the immunostimulatory RNAs are generated during DNA virus infection. Now, we know these RNAs could be the transcripts either from cellular or foreign genomes.

In physiological conditions, cytoplasm DNA existence should be avoided. Nevertheless, under some circumstances, such as autoimmune responses or virus infection, host DNA and/or foreign DNA would be inappropriately delivered into the cytosol, and this DNA could be transcribed by cytoplasmically localized Type III RNA polymerase (RNAPIII) (Chiu et al., 2009). Though three main RNA polymerases are expressed in eukaryotic cells, only type III is abundant in the cytosol. RNAPIII is also the largest RNA polymerase currently known and consists of 17 subunits, including DNA binding sites, which can catalyze gene transcription (Hoffmann et al., 2015). Initially, RNAPIII was reported as a sensor for cytoplasmic DNA during DNA virus infection (Ablasser et al., 2009; Luke et al., 2011). It can also transcribe short-spread and repetitive elements in the human genome. During innate immune response, RNAPIII synthesizes 5S rRNA, tRNA, and other small RNAs through a specific promoter region, taking the dsDNA-rich AT as a template to transcribe into 5′-triphosphate dsRNA, which is the RIG-I ligand (Ablasser et al., 2009; Chiu et al., 2009; Luke et al., 2011). Likely, the viral genome containing the AT-rich features could induce RNAPIII-dependent RNA synthesis (Ablasser et al., 2009; Chiu et al., 2009). Consistent with this, AT-rich DNA sequences could be transcribed during ionizing radiation treatment and initiate MAVS-dependent RNA sensing and signaling (Feng et al., 2020).

In addition to being present and functioning in the cytosol, RNAPIII is also a nucleus resident who transcribes RNAs to activate RLR/MAVS signaling, as illustrated during Epstein-Barr virus (EBV) infection (Rosa et al., 1981). After infection, the virus establishes life-long latency in the host cell, and only a small set of viral transcripts are produced. EBV-encoded small RNAs (EBERs) transcribed by RNAPIII, which consists of EBER1 and EBER2, are the most abundant viral transcripts during EBV latency. These EBERs contain biochemical features that could be recognized by RIG-I and are sufficient to activate RLR/MAVS signaling (Glickman et al., 1988; Samanta et al., 2006, 2008). Furthermore, the exosome-dependent transition of EBER1 was identified. EBER1 could be sensed by both TLR-3 and RIG-I in target cells, which is also dependent on the presence of its 5′-triphosphate moiety (Iwakiri et al., 2009; Baglio et al., 2016).

The importance of RNAPIII in controlling varicella-zoster virus (VZV) infection is revealed in a study showing that mutation in the RNAPIII machinery results in severe illness (Ogunjimi et al., 2017). Furthermore, inhibition of RNAPIII with ML-60218 reduced IFN-β production during HSV-1, EBV, and adenovirus infection, but not an RNA virus, Sendai virus infection (Chiu et al., 2009). Virus-associated RNAs (VAs) were confirmed in Adenovirus infection for RIG-I downstream signaling activation. These small non-coding RNAs are highly similar to the EBV-encoded EBERs (Rosa et al., 1981). Whether the similar RNAs activate the signaling in HSV infection needs further investigation. The expression of type I interferon induced by adenovirus VAs also coincides with the VA-RNA expression phase during the virus infection (Minamitani et al., 2011). Furthermore, it seems that VA functions differently in different cell types. The production of type I IFN in MEFs was completely dependent on RLR/MAVS signaling, whereas, in generated bone marrow-derived dendritic cells (GM-DCs) it was partially dependent (Yamaguchi et al., 2010). Since adenovirus vector is used widely in fundamental research and clinical practice, these studies should advance the vector designs (Machitani et al., 2011; Darweesh et al., 2019).

Aside from the immunostimulatory RNAs transcribed in the cytoplasm, transcripts of retrotransposons and pseudogenes from the host genome that can activate RLR/MAVS have also been identified in DNA virus infection. During murine gammaherpesvirus 68 (MHV-68) infection, SINE (short interspersed nuclear element) RNAs are rapidly transcribed by RNAPIII in the nucleus and activate the antiviral NF-κB signaling pathway through MAVS-dependent and independent mechanisms (John et al., 2015). HSV-1 infection induces relocalization of 5S ribosomal RNA pseudogene 141 (RNA5SP141) from the nucleus to the cytoplasm that binds to RIG-I and activates the downstream signaling (Kang et al., 2004). HSV-1 infection could induce the shutoff of host protein synthesis, such as ribosomal protein L5 (RPL5). RPL5 associates with 5S rRNA to form 5S ribonucleoprotein particles. Both mitochondrial ribosomal protein L18 (MRPL18) and thiosulfate sulfurtransferase (TST) facilitate the import of cytoplasmic 5S rRNA into mitochondria (Zhang et al., 2007; Smirnov et al., 2010, 2011). HSV-1-mediated shutoff of these protein synthesis could liberate and relocate RNA5SP141 in cytoplasm for recognition by RIG-I (Chiang J.J. et al., 2018).

Vault RNAs (vtRNAs) are another type of host small RNAs. They initially contain a 5′-triphosphate after being transcribed by RNAPIII, and the 5′-triphosphate moieties are removed by the cellular triphosphatase, dual-specificity phosphatase 11 (DUSP11), in uninfected cells resulting in the conversion of these RNAs to be non-immunostimulatory (Zhao et al., 2018). During KSHV infection, the expression of this type of RNAs are enhanced. Furthermore, KSHV infection reduces the expression of host DUSP11, which leads to the accumulation of immunostimulatory RNAs (Nottingham et al., 2016; Yang et al., 2018). A recent study also showed that transcripts from multiple KSHV genomic regions bind to and activate RIG-I during the virus infection (Zhang et al., 2018).

## Evasion of the RLR Signaling Pathway by DNA Viruses

Although RLR-mediated innate immune response could elicit a robust immune response against incoming DNA viruses, these viruses have adopted multiple evasion mechanisms for counteracting and escaping such immune responses. RLR recognition of PAMPs, responsible for initiating antiviral signaling, is a target for these viruses. Damping of RLRs and host factors participating in RLR activation regulation in the signaling pathway is one of the key mechanisms for these viruses’ evasion. For example, EBV can promote RIG-I degradation through the proteasomal pathway by recruiting an E3 ubiquitin ligase, the carboxyl-terminus of Hsp70 interacting protein (CHIP) to the RIG-I through EBV-encoded LMP1 (Chongfeng et al., 2018). Our previous studies found that human herpesvirus 6 (HHV-6), a DNA virus, can regulate the RLR/MAVS signaling pathway by affecting the mitochondrial membrane potential (Jiang et al., 2021). Here, we summarize the strategies that DNA viruses employed to evade the RLRs-mediated innate antiviral responses. Eliminating the sensor or a factor in the signaling pathway is one of the effective mechanisms to suppress the immune responses. Many viruses encode viral proteases that directly cleave RLR, as mentioned above of EBV infection (Baglio et al., 2016). Since MAVS is critical for both RIG-I- and MDA5-mediated signaling, many viral products, such as HCMV US9 and HHV-6 U26 proteins, target and cleave MAVS by inducing the leakage of mitochondrial MAVS for degradation (Choi et al., 2018; Jiang et al., 2021). Moreover, during HCMV infection RIG-I was significantly degraded (Scott, 2009). Hepatitis B virus (HBV) also produces RNA species during its life cycle, which binds to MAVS and promotes its degradation through Lys136 ubiquitination (Sato et al., 2015). A human herpesvirus 8 (HHV-8) gene product, viral interferon regulatory factor 1 (vIRF-1) is targeted to the mitochondrial detergent-resistant membrane (mDRM) during virus replication. It was shown that vIRF-1 negatively regulates the MAVS-mediated antiviral responses (Lin et al., 2001; Hwang and Choi, 2016). And the interaction of HSV-1 US11 protein with RIG-I and MAVS results in the impediment of RIG-I/MAVS and MDA5/MAVS complex formation and subsequent reduced production of IFN-β (Xing et al., 2012; Su et al., 2016; Zhu and Zheng, 2020). Other strategies may also be taken by HSV-1 for the evasion of RLR detection (Wang et al., 2013b) and the subsequent signaling (Zhu et al., 2011; Wang et al., 2013c,^2014^; Xing et al., 2013; Zhang et al., 2013).

Manipulation of post-translational modification of RLRs is another key strategy for immune evasion. As described above, reversed ubiquitination is an important step in regulating RLR signaling cascade. Many DNA viruses attenuate IFN response by altering the ubiquitination of RLRs and/or RLR-regulatory proteins through various mechanisms. Since the E3 ligase TRIM25 is a key activator of the RIG-I-mediated signaling pathway (Castanier et al., 2012), many viruses, including DNA viruses, target this protein for immune evasion. During human papillomavirus (HPV) infection, the E6 oncoprotein interacts with TRIM25 and ubiquitin-specific peptidase 15 (USP15), which enhances TRIM25 degradation and subsequently inhibits RLR/MAVS signaling. These interactions promote the ubiquitination and degradation of TRIM25. Moreover, these processes inhibit the ubiquitination associated with RIG-I and K63, block RIG-I-MAVS interactions, and ultimately prevent the activation of RIG-I-mediated signaling (Chiang C. et al., 2018). KSHV encodes a viral deubiquitinating enzyme (DUB), ORF64. This viral enzyme effectively removes both K48-linked and K63-linked ubiquitin chains from RIG-I, which inhibits the activation of RLR/MAVS signaling (González et al., 2009; Inn et al., 2011). DNA viruses contain a relatively large genome compared with RNA viruses. There should be no doubt that other strategies, such as manipulating cellular DUB expression, used by many RNA viruses, would also be used by DNA viruses.

## Concluding Remarks and Future Perspectives

**FIGURE 1:**
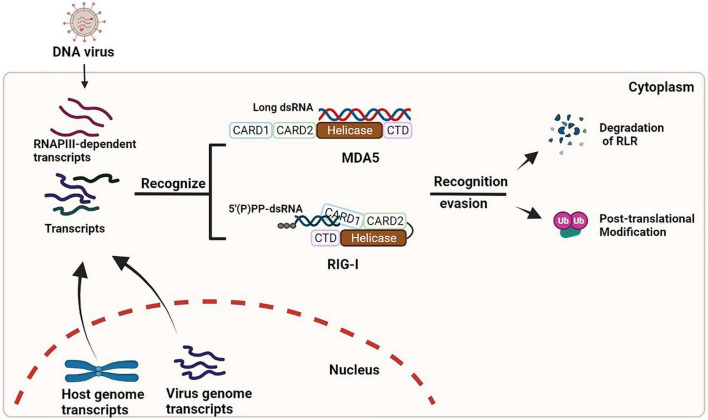
Activation and evasion of RLR signaling during DNA virus infection. During infection of many DNA viruses, some viral transcripts and/or cellular RNAs synthesized by RNAPIII can be detected by RLRs, which results in the activation of RLR signaling (left). These viruses also have developed multiple strategies (such as degradation and/or modification of RLRs) to dampen the signaling (right).

In recent years, the roles of activating the RLR/MAVS signaling pathway during DNA virus infection has been identified. However, the genome features of these DNA viruses are not readily for RLR recognition. Some of the viral transcripts and the unusual cellular RNAs (mislocated or unmarked) can be recognized and bound by RLRs, which could subsequently trigger an antiviral innate immune response that is proven to play an important role in controlling viral infections. Meanwhile, almost all these viruses have also developed individual strategies to escape RLR/MAVS-mediated antiviral response, which needs to be elucidated in the contexts of each virus infection (summarized in Figure 1).

A large amount of studies in this field has been focused on elucidating mechanisms of immunostimulatory RNA ligands for RLR recognition, modification of key components of the RLR/MAVS pathway, and the evasion strategies usurped by the viruses. Additional studies for most DNA viruses have to be conducted to investigate whether RLR/MAVS signaling activation is a general phenomenon and the physiological roles of the signaling in the disease contexts. Furthermore, it is worth to clarifying whether and how RLR/MAVS signaling cooperates with other innate immune signaling.

## Author Contributions

All authors listed have made a substantial, direct, and intellectual contribution to the work, and approved it for publication.

## Conflict of Interest

JF is employed by Genor Biopharma Co., Ltd. The remaining authors declare that the research was conducted in the absence of any commercial or financial relationships that could be construed as a potential conflict of interest.

## Publisher’s Note

All claims expressed in this article are solely those of the authors and do not necessarily represent those of their affiliated organizations, or those of the publisher, the editors and the reviewers. Any product that may be evaluated in this article, or claim that may be made by its manufacturer, is not guaranteed or endorsed by the publisher.
